# Evaluation of Three Culture Media for Isolation of *Burkholderia cepacia* Complex from Respiratory Samples of Patients with Cystic Fibrosis

**DOI:** 10.3390/microorganisms9122604

**Published:** 2021-12-16

**Authors:** Emma C. L. Marrs, Audrey Perry, John D. Perry

**Affiliations:** Microbiology Department, Freeman Hospital, Newcastle upon Tyne NE7 7DN, UK; Emma.marrs@nhs.net (E.C.L.M.); audrey.perry@nhs.net (A.P.)

**Keywords:** *Burkholderia cepacia* complex, cystic fibrosis, culture media

## Abstract

*Burkholderia cepacia* complex (BCC) is a significant pathogen causing respiratory disease in individuals with cystic fibrosis (CF). Diagnosis is typically achieved by isolation of BCC on selective culture media following culture of sputum or other respiratory samples. The aim of this study was to compare the efficacy of three commercially available selective media for the isolation of BCC. The three media comprised Burkholderia cepacia selective agar (BCSA; bioMérieux), BD Cepacia medium (BD: Becton–Dickinson) and MAST Cepacia medium (MAST laboratories). Each medium was challenged with 270 respiratory samples from individuals with CF as well as an international collection of BCC (*n* = 26) and 14 other isolates of *Burkholderia* species at a range of inocula. The international collection was also used to artificially “spike” 26 respiratory samples. From a total of 34 respiratory samples containing BCC, 97% were recovered on BD and 94% were detected on MAST and BCSA. All three media were effective for isolation of BCC. BCSA was much more selective than the other two media (*p* < 0.0001) meaning that fewer isolates required processing to exclude the presence of BCC.

## 1. Introduction

Individuals with cystic fibrosis (CF) are susceptible to respiratory infections caused by non-fermentative Gram-negative bacteria. Among such bacteria, *Burkholderia cepacia* complex (BCC) has the most deleterious impact in terms of morbidity and mortality. Qvist et al. examined the impact of a range of pathogens on the lung function of 432 patients with CF in a longitudinal registry study spanning 40 years [1]. They showed that, among Gram-negative pathogens, BCC had the greatest negative impact on lung function and was second only to *Mycobacterium abscessus* complex in terms of severity. In the context of lung transplantation, BCC is particularly problematic and is associated with the development of “cepacia syndrome”, a necrotising pneumonia often leading to sepsis with high rates of mortality [2].

The BCC consists of at least 22 closely related species [3] and certain species are more pathogenic than others in the context of CF [4]. Post lung transplantation, *Burkholderia cenocepacia* is particularly associated with an increased rate of mortality [5,6] and, in many centres, infection with *B. cenocepacia* excludes the possibility of lung transplantation in favour of recipients more likely to have a successful outcome [7]. The prevalence of BCC among individuals with CF has declined to around 3% in Europe according to data from the European CF Society patient registry [8]. This decline is due to improved infection control measures to reduce patient-to-patient transmission of BCC, including the segregation and cohorting of infected individuals within clinics [9].

Detection of BCC in individuals with CF is typically achieved by routine culture of sputum and other respiratory samples onto highly selective agar-based culture media [10]. This high selectivity, due to the incorporation of antimicrobial agents, is essential to eliminate the growth of other Gram-negative bacteria (e.g., *Pseudomonas aeruginosa*) and fungi (e.g., *Aspergillus fumigatus*) that are commonly found in the sputum of individuals with CF and may overgrow BCC.

The aim of this study was to compare three selective media that are commercially available in the United Kingdom for the isolation of BCC. This was achieved by challenging each of the media with an international collection of BCC using a range of different inocula and also by culture of respiratory samples from individuals with CF.

## 2. Materials and Methods

### 2.1. Culture Media

Burkholderia cepacia selective agar (BCSA; product ref: 33631) was obtained from bioMérieux, Craponne, France, as pre-poured plates. BD Cepacia medium (product ref: 256180) was obtained from Becton-Dickinson, Rungis, France as pre-poured plates. MAST Cepacia medium was prepared weekly in our laboratory using materials purchased from MAST Diagnostics Ltd., Bootle, UK. Preparation was performed in exact accordance with manufacturer’s instructions using dehydrated B. cepacia medium (Product ref: DM253D;) supplemented at 45 °C with B. cepacia Selectavial (Product code: SV-22). MAST cepacia medium was used within 7 days of preparation and other media were used before their specified expiry date. Columbia blood agar (Oxoid, Basingstoke, UK) was used for general subcultures.

### 2.2. Bacterial Strains

Strains used for quality control of culture media were obtained from the National Collection of Type Cultures (NCTC), Colindale (London), UK. Twenty-six strains of BCC were obtained from the Belgium Coordinated Collections of Microorganisms/Laboratorium Microbiologie Ghent (BCCM/LMG) at the University of Ghent, Belgium. These strains were selected from a diverse international collection [11,12]. A further 14 isolates of *Burkholderia* spp., including 13 BCC and 1 *Burkholderia gladioli*, were retrieved from the stored collection of the Freeman Hospital Microbiology Department. All of these 14 isolates were recovered in previous years from distinct patients with CF. In total, 40 isolates of *Burkholderia* spp. were tested including: *Burkholderia ambifaria* (*n* = 2), *Burkholderia anthina* (*n* = 2), *Burkholderia cenocepacia* (*n* = 8), *Burkholderia cepacia* (*n* = 3), *Burkholderia contaminans* (*n* = 2), *Burkholderia dolosa* (*n* = 2), *Burkholderia gladioli* (*n* = 1), *Burkholderia multivorans* (*n* = 10), *Burkholderia pyrrocinia* (*n* = 2), *Burkholderia seminalis* (*n* = 1), *Burkholderia stabilis* (*n* = 3) and *Burkholderia vietnamiensis* (*n* = 4).

### 2.3. Quality Control of Culture Media

Each batch of media was challenged with approx. 10^5^ colony-forming units (CFU) of the following strains *B. cepacia* NCTC 10743, *Escherichia coli* NCTC 12241, *P. aeruginosa* NCTC 12903, *Stenotrophomonas maltophilia* NCTC 10257 and *Staphylococcus aureus* NCTC 12973. This was achieved by using 1 µL of a 0.5 McFarland suspension prepared using a Densimat (bioMérieux, Basingstoke, UK), from a fresh overnight culture on Columbia blood agar. After 48 h incubation at 37 °C, only *B. cepacia* was able to grow on each of the three selective media.

### 2.4. Challenge Experiments Using Pure Strains of Burkholderia Species

Strains from the international collection of BCC (*n* = 26) were supplemented with clinical isolates of *Burkholderia* species from the Freeman Hospital collection (*n* = 14) to challenge each of the three media using various inoculum levels. This was performed to assess the sensitivity of the media for supporting the growth of *Burkholderia* species. Each strain was subcultured onto Columbia blood agar and incubated for 24 h at 37 °C. A suspension was then prepared with turbidity equivalent to 0.5 McFarland units using a Densimat. Serial tenfold dilutions (1/10–1/10,000) were then performed by adding 100 µL of suspension to 0.9 mL of sterile saline (0.85%). This was intended to generate a series of inocula ranging from approx. 10^8^–10^4^ CFU/mL. A 1 µL aliquot of each suspension was then inoculated onto each of the three test media, as well as Columbia blood agar, using a multipoint inoculation device (Mast Diagnostics Ltd., Bootle, UK). Each type of medium was inoculated in triplicate and strains were only regarded as being “detected” on a particular medium if growth was consistently present on all three replicates. Plates were examined after 24, 48 and 72 h of incubation at 37 °C.

To validate the inoculum for each strain, the suspension expected to contain approx. 10^4^ CFU/mL was sampled by removal of 10 µL, which was then inoculated onto Columbia blood agar and spread across the whole surface of the plate. Plates were incubated at 37 °C for 72 h. These counts were performed in duplicate and mean colony counts were calculated.

### 2.5. Culture of Clinical Samples

Over a seven-month period, respiratory samples were referred to the Freeman Hospital Microbiology Department from 270 distinct patients who were attending specialised clinics for individuals with CF. The samples included 171 cough swabs, 97 sputum samples and 2 samples of broncho-alveolar lavage (BAL). The specimens were collected as part of routine monitoring of the patients and no specimens were collected for the purposes of this study. As part of routine processing, sputum samples were treated with an equal volume of sputasol (Product SR0233A; Oxoid, Basingstoke, UK) and mixed thoroughly using a vortex mixer until homogeneous. All respiratory samples were then cultured for a range of pathogens in line with routine laboratory procedures. Leftover aliquots of these samples were then anonymised by laboratory staff and processed as described below.

Material from each cough swab was suspended in 1 mL of sterile physiological saline (0.85%) and, after vortexing for 30 s, a 50 µL aliquot was inoculated onto BCSA, MAST cepacia medium and BD cepacia medium. For homogenised sputum samples, a 50 µL aliquot was inoculated onto each of the same three media. Finally, for BAL, a 50 µL aliquot was inoculated onto each culture medium, without the use of sputasol. The inocula on the three culture media were spread to obtain isolated colonies and all media were incubated at 37 °C for 72 h. All three media were examined and colonies investigated after 24, 48 and 72 h (see Section 2.7). MAST plates were then examined after an additional 5 days at room temperature in accordance with manufacturer’s instructions.

### 2.6. Culture of “Spiked” Clinical Samples

To artificially increase the number of positive samples, 26 samples (18 sputa and 8 cough swabs) were each “spiked” with a unique strain of BCC from the international collection. Each strain was subcultured onto Columbia blood agar and incubated for 24 h at 37 °C. A suspension was then prepared with turbidity equivalent to 0.5 McFarland units using a Densimat. A 1/100 dilution was then performed by adding 10 µL of this suspension to 990 µL of sterile saline (0.85%). Ten microlitres of this diluted suspension were then added to either 1 mL of homogenised sputum or 1 mL of cough swab suspension. A 50 µL aliquot of each “spiked” sample was then cultured exactly as described above. The estimated inoculum of BCC delivered onto each plate was approx. 250–500 CFU.

### 2.7. Identification

All bacteria and yeasts recovered on all media were subcultured onto Columbia blood agar, incubated overnight at 37 °C and subsequently identified using matrix-assisted laser desorption/ionisation-time of flight mass spectrometry (MALDI-TOF MS) in accordance with manufacturer’s instructions (Bruker, Coventry, UK). A bacterial test standard supplied by the manufacturer was used for daily calibration of the instrument and a collection of NCTC control strains representing 14 different bacteria and yeasts were tested weekly as part of routine quality control, as recommended by UK national standards [13].

Isolates of filamentous fungi were assigned to genus or species level based on macroscopic and microscopic appearance.

### 2.8. Statistical Analysis

Each culture medium was compared with each other medium using McNemar’s test with the continuity correction applied. Statistical significance was taken as *p* < 0.05.

## 3. Results

### 3.1. Detection of Pure Isolates of Burkholderia Species at a Range of Inocula

All three media were challenged with 40 isolates that included the international collection of BCC (*n* = 26) and 14 clinical strains of *Burkholderia* spp. (*n* = 14). The average viable count for a 0.5 McFarland standard for these 40 isolates was approximately 6 × 10^7^ CFU/mL (range: 2.1 × 10^7^ to 1.1 × 10^8^ CFU/mL). Consequently, the plates were challenged with the following average inocula: 60,000; 6000; 600; 60 and 6 CFU/spot. Unsurprisingly, there was some variability between replicates on all media when a very low inoculum of six CFU/spot was tested, but no variability across replicates when higher inocula were used.

**Figure 1:**
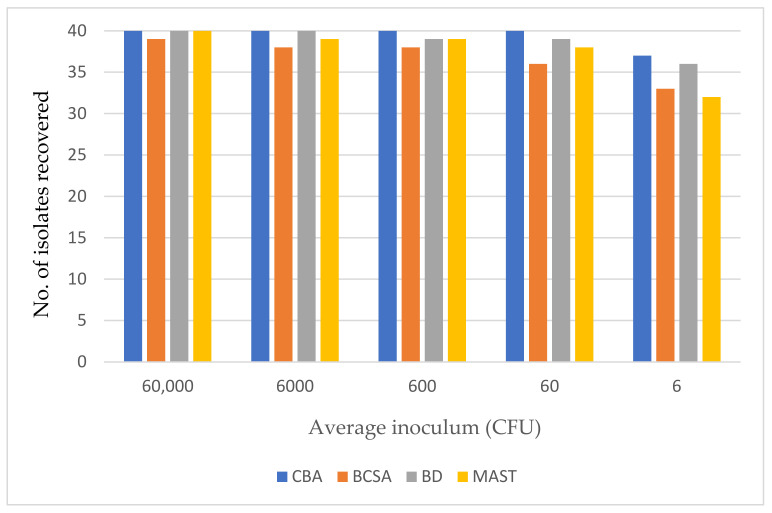
The effect of inoculum on the recovery of *Burkholderia* species on three selective media and Columbia blood agar (CBA) after 72 h incubation.

Using a high inoculum of approx. 60,000 CFU/spot, all isolates were recovered on the three selective media except for *B. ambifaria* (LMG 19467) which was inhibited by BCSA and failed to grow. Figure 1 shows the effect of inoculum on the recovery of *Burkholderia* species on three selective media.

### 3.2. Detection of Burkholderia cepacia Complex in Clinical Samples

Using a combination of all media, BCC were recovered from only 8 out of the 270 samples (3%). This is consistent with data from the European CF Society patient registry, which indicate a prevalence of around 3% in Europe [8]. All eight strains were recovered on MAST and BCSA within 72 h. One strain of *B. multivorans* was not recovered on BD; however this strain only produced 1–2 colonies on the other two media, suggesting that this failure may have been attributable to chance.

Due to the low positivity rate, it was necessary to use artificially “spiked” samples. Twenty-six samples (from which no BCC was recovered on any medium in the absence of “spiking”) were artificially inoculated with 26 unique strains of BCC from an international collection. All 26 strains were recovered on BD medium within 72 h whereas two isolates were not recovered on MAST medium and BCSA. As predicted from the results in Section 3.1, *B. ambifaria* (LMG 19467) was not recovered on BCSA from “spiked” sputum but this strain was recovered in high counts (>100 colonies) on the other two selective media. Another isolate that was not recovered on BCSA when “spiked” into a cough swab was *B. multivorans* (LMG 13010). This isolate grew very poorly on BCSA when challenged with 600 CFU/spot of the pure culture and was completely inhibited at ≤60 CFU/spot.

Two strains from the international collection remained undetected from culture of “spiked” samples on MAST medium. The first strain, *B. multivorans* (LMG 17588), also failed to grow on MAST medium when tested as a pure culture at an inoculum of ≤600 CFU/spot. The second strain, *B. anthina* (LMG 20980), remained undetected in a culture that showed heavy growth of *P. aeruginosa*.

**Figure 2:**
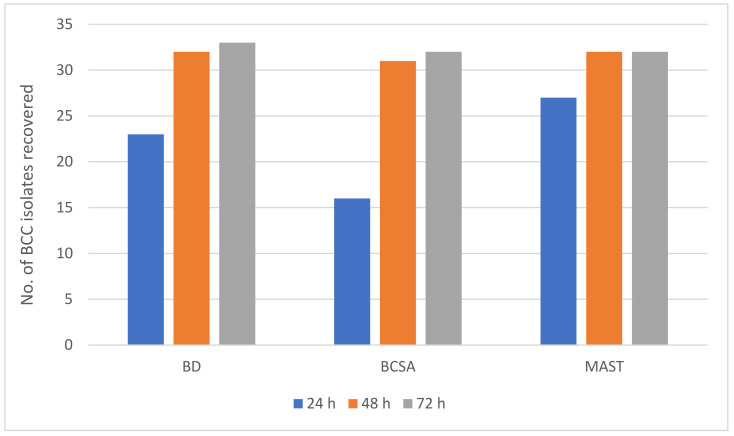
Number of BCC isolates recovered on three selective media from 270 respiratory samples and 26 spiked samples over the 72 h incubation period.

There was evidence of delayed growth on BCSA when compared with the other two media with fewer isolates detected after 24 h of incubation. Where isolates were recovered on BCSA after 24 h, the colony size was invariably smaller when compared with colonies on the other two media. Figure 2 summarises data from a combination of natural and “spiked” samples. It is clear from Figure 2 that the three media recovered an almost identical number of BCC strains within 72 h.

### 3.3. Comparison of the Selectivity of Three Selective Media

Table 1 summarises the recovery of all non-*Burkholderia* species that were recovered on the three selective media from 270 respiratory samples. To avoid counting the same isolates twice, isolates that were recovered from spiked samples are not included in the table. Over the 72 h incubation period, 40 non-*Burkholderia* isolates were recovered from 270 respiratory samples on BCSA. The other two media were significantly less selective with 80 isolates recovered on BD medium and 108 isolates recovered on MAST. Among Gram-negative species, *Achromobacter xylosoxidans* was recovered more frequently on BCSA but fewer isolates of *P. aeruginosa* were able to grow. BCSA was substantially more inhibitory for yeasts and Gram-positive bacteria than the other two media.

The likelihood of isolating a non-*Burkholderia* from a clinical sample was significantly lower on BSCA compared with either of the other two media (*p* < 0.0001). The likelihood of isolating a non-*Burkholderia* from a clinical sample was significantly lower on BD compared with MAST (*p* = 0.04).

BD offered the best fertility of the three selective agars with all 40 isolates recovered at an inoculum of ≥6000 CFU/spot and 36/40 isolates consistently detected (across three replicates) with a very low inoculum of approx. 6 CFU/spot—only one isolate fewer than detected by Columbia blood agar. The manufacturer’s instructions for MAST recommend an additional 5 days’ incubation at room temperature after 48 h incubation at 35–37 °C. This had no impact on the recovery of pure isolates of BCC at various inocula and had no impact on the isolation of BCC from clinical samples. 

## 4. Discussion

The first published reports to describe specific culture media for isolation of BCC from sputum samples appeared in the mid-1980s. Gilligan et al. [14] described a new medium designated “PC medium” that utilised crystal violet, bile salts, ticarcillin and polymyxin B as selective agents and demonstrated superior recovery of BCC when compared to general purpose media such as MacConkey agar. Welch et al. [15] reported similar findings with a medium they designated “OFPBL” that employed polymyxin B and bacitracin as selective agents as well as lactose with a pH indicator to provide some differentiation of BCC from other flora. Laboratory proficiency testing reported by Tablan et al. [16] showed that 95% of laboratories that used a dedicated selective medium (PC medium or OFPBL) were able to recover BCC from simulated sputum samples, compared with 22% of laboratories who relied on general purpose media. Several years later, Henry et al. [17] described the development of BCSA that utilised polymyxin, gentamicin and vancomycin as selective agents along with lactose, sucrose and phenol red to help differentiate BCC. These formulations form the basis of the selective media that are currently commercially available for isolation of BCC, with MAST medium and BD medium utilising the selective agents used in PC medium and bioMérieux utilising a formulation based on BCSA [18].

Few studies have compared selective media for BCC using sputum samples from individuals with CF. In the largest study to date, Henry et al. compared PC medium with OFPBL and BCSA for the isolation of BCC from 656 respiratory samples [19]. They reported 100% sensitivity for BCSA after 72 h incubation compared with 96 and 84% for OFPBL and PC medium, respectively. BCSA was found to be substantially more selective than the other two media. Wright et al. compared BCSA with the selective medium available from MAST Laboratories using 149 sputum samples. They also found 100% sensitivity within 72 h for BCSA compared to 94% for the medium from MAST. They confirmed the higher selectivity of BCSA [20].

In this study, there was also a clear difference between BCSA and the other two media in terms of selectivity. BCSA is much more selective—particularly against Gram-positive bacteria, yeasts and fungi and also *Pseudomonas aeruginosa*—which is very common in CF respiratory samples. This high selectivity has a small negative effect on the growth of some strains of BCC. Firstly, there is a delayed detection of growth with a lower yield at 24 h on BCSA when compared with the other two media. Secondly, there is evidence that occasional strains (such as *B. ambifaria* LMG 19467) may be completely inhibited even when a high inoculum is used (60,000 CFU/spot). This is only likely to be a significant problem if such a strain is responsible for an outbreak. It is important to note that culture for BCC is invariably carried out alongside culture for other CF pathogens using less selective media that can also potentially recover BCC. Others, including van Pelt et al. [21], have emphasised the need to use a combination of selective and non-selective media for isolation of BCC. This difference in selectivity is explained by the choice of selective agents. All three media utilise polymyxin B and crystal violet but media from MAST and BD utilise bile salts and ticarcillin, whereas BCSA contains gentamicin and vancomycin [18]. Inclusion of vancomycin in BCSA likely explains its higher selectivity against Gram-positive bacteria.

High selectivity can be an advantage in the context of CF, where pathogens can easily be overgrown by other bacteria or fungi. In one example in this study, it seems very likely that a heavy growth of *P. aeruginosa* on MAST prevented isolation of a smaller amount of BCC. Furthermore, it is clear that a lot of labour time can be saved when processing cultures on BCSA due to the much lower number of non-*Burkholderia* species that are recovered. If any colony is regarded as potential BCC then the positive predictive value (PPV) of BCSA was 17%, compared with 8% for BD and 7% for MAST. No attempt was made to assess the PPV based on colony colour, as the colour of BCC proved to be highly variable, partly due to the production of natural pigments by some strains. For example, on MAST medium, BCC strains from the international collection generated colonies described as pink, purple, green and yellow. Similarly, the Type strain of *B. cepacia* (NCTC 10743) generates green colonies on BCSA whereas most other isolates of BCC produce purple colonies.

Other methods have been explored for detection of BCC in sputum samples including PCR-based methods, with some evidence that BCC can be detected when cultures are negative, possibly due to a low bacterial load [22,23]. In order to increase the sensitivity of culture, others have exploited the use of selective enrichment broths prior to culture on agar-based media with conflicting results reported [24,25].

## 5. Conclusions

For 34 samples that were naturally or artificially colonised with a diverse collection of BCC, BCSA showed the same sensitivity as MAST (94%), with BD recovering one additional isolate (sensitivity: 97%). All three media are effective for recovery of BCC from respiratory samples from patients with CF. BCSA offers a higher selectivity, meaning that fewer isolates require processing, thus potentially allowing for savings to be made in terms of labour time and reagents for bacterial identification. Our findings endorse those of Henry et al., who also confirmed the high sensitivity combined with high selectivity afforded by BCSA [19].

## Figures and Tables

**Table 1:** A summary of all non-*Burkholderia* isolates recovered by culture of 270 respiratory samples on three selective culture media after 72 h incubation.

	BD	BCSA	MAST
**Gram-negative species**	**35**	**32**	**38**
*Achromobacter* species	2	2	1
*Achromobacter xylosoxidans*	7	17	8
*Chryseobacterium* species	2	3	3
*Cupriavidus metallidurans*	0	1	0
*Klebsiella pneumoniae*	1	0	2
*Morganella morganii*	0	0	1
*Pandoraea* species	1	2	1
*Proteus mirabilis*	1	0	1
*Pseudomonas aeruginosa*	13	4	13
*Pseudomonas fluorescens* group	2	0	3
*Pseudomonas* species (other)	1	0	2
*Sphingomonas* species	1	2	1
*Stenotrophomonas maltophilia*	4	1	2
**Gram-positive species**	**17**	**1**	**36**
*Enterococcus faecalis*	0	1	1
*Enterococcus faecium*	5	0	5
*Enterococcus raffinosus*	1	0	1
*Lactobacillus rhamnosus*	0	0	1
*Mycobacterium abscessus* complex	3	0	3
*Nocardia criacigeorgica*	1	0	1
*Staphylococcus aureus*	1	0	1
*Staphylococcus epidermidis*	5	0	16
*Staphylococcus haemolyticus*	1	0	5
*Streptococcus salivarius*	0	0	2
**Yeasts and fungi**	**28**	**7**	**34**
*Aspergillus fumigatus*	15	2	18
*Candida albicans*	0	1	0
*Candida parapsilosis*	11	4	13
*Candida* species (other)	1	0	1
*Scedosporium apiospermum*	1	0	2
**Total**	**80**	**40**	**108**

## Data Availability

All relevant data is included in the manuscript.
